# A multimodal ConvNeXt-Tiny deep learning model for simultaneous prediction of IDH mutation and Ki-67 expression in gliomas

**DOI:** 10.1371/journal.pone.0351757

**Published:** 2026-06-26

**Authors:** Juan Du, Linsha Yang, Duo Zhang, Jinguo Wang, Shuo Wu, Defeng Liu, Huiling Yi, Xin Liang, Xiaohan Wang, Qinglei Shi, Tao Zheng

**Affiliations:** 1 Department of Medical Imaging, The First Hospital of Qinhuangdao, Qinhuangdao, Hebei Province, China; 2 Department of Medical Imaging, Baoding No. 1 Central Hospital, Baoding, Hebei Province, P.R. China; 3 Department of Magnetic Resonance Imaging, Qinglong Manchu Autonomous County Traditional Chinese Medicine Hospital, Qinglong County, Qinhuangdao, Hebei Province, China; 4 Scientific Clinical Specialist, Siemens Healthineers China, Beijing, China; Università degli Studi di Napoli Federico II: Universita degli Studi di Napoli Federico II, ITALY

## Abstract

**Objective:**

To construct and validate a multi-task deep learning model based on ConvNeXt-Tiny for synchronous prediction of isocitrate dehydrogenase (IDH) mutation status and Ki-67 expression level in gliomas.

**Materials and Methods:**

This retrospective multicenter study included adult patients with diffuse glioma from two hospitals, which served as the training set and the independent validation set, respectively. All patients underwent multimodal MRI examinations within 4 weeks before surgery, including T2-weighted imaging (T2WI), T2-fluid attenuated inversion recovery (T2-FLAIR), contrast-enhanced T1-weighted imaging (T1CE), and three-dimensional arterial spin labeling imaging (ASL). MRI data were uniformly preprocessed to extract traditional radiomics features, and a dual-task deep learning model based on ConvNeXt-Tiny was constructed. Clinical characteristics, radiomics features, and deep learning features were multimodally fused to establish the multimodal model and were compared with other prediction models. Model performance and clinical utility were evaluated using receiver operating characteristic (ROC) curves, calibration curves, decision curve analysis, and the net reclassification index (NRI). SHapley additive explanations (SHAP) analysis and gradient-weighted class activation mapping (Grad-CAM) were applied for visual interpretation of model decisions.

**Results:**

The multimodal model demonstrated the best diagnostic performance for both IDH mutation status and Ki-67 expression level prediction. For IDH prediction, the multimodal model achieved AUCs of 0.901 and 0.883 in the training set and test set, respectively, while for Ki-67 prediction, the area under the curves (AUCs) reached 0.939 and 0.924. NRI analysis further confirmed that the multimodal model significantly improved case reclassification in both tasks. Calibration curves and the Hosmer–Lemeshow test indicated good model fit, and decision curve analysis showed a higher net clinical benefit. SHAP analysis revealed that model predictions mainly relied on deep learning features, particularly those derived from T1CE, ADC, and cerebral blood flow (CBF) images, whereas radiomics features and clinical variables contributed less. Grad-CAM visualizations demonstrated that the model primarily focused on tumor-related regions.

**Conclusion:**

This study developed a ConvNeXt-Tiny-based multi-task model that enables preoperative synchronous prediction of IDH mutation and Ki-67 status in adult diffuse glioma and exhibits robust performance superior to single-task and traditional models.

## Introduction

Glioma is the most common primary malignant tumor of the central nervous system, accounting for approximately 30% of all brain tumors and 80% of all malignant tumors. Its incidence is about 6–7 cases per 100,000 people per year and is rising globally [1]. Simple histological grading is no longer sufficient to fully describe the high heterogeneity of gliomas [2]. This heterogeneity is reflected not only in cell morphology and proliferative activity, but more profoundly at the molecular level, including driver gene mutations, epigenetic regulation, and the tumor microenvironment. These factors directly lead to marked differences in treatment response and prognosis among patients receiving standard therapies [3]. The isocitrate dehydrogenase (IDH) mutation status and the Ki-67 proliferation index are two key biomarkers with decisive clinical significance. IDH-mutant gliomas are associated with significantly longer overall survival and better sensitivity to chemotherapy, whereas IDH wild-type gliomas are more aggressive and have a poorer prognosis [4]. Ki-67, as a direct indicator of cellular proliferative activity, is closely related to tumor growth rate, invasiveness, and recurrence risk, and serves as an important marker for evaluating malignancy, predicting treatment response, and monitoring disease progression [5]. These biomarkers offer complementary value for individualized treatment planning [5]. However, postoperative histopathological assessment of IDH and Ki-67 is invasive and prone to sampling bias due to intratumoral heterogeneity [6,7]. Therefore, a non-invasive and repeatable preoperative approach for their simultaneous assessment is critically needed.

Magnetic resonance imaging (MRI), owing to its non-invasive nature, has become the primary modality for preoperative molecular marker prediction. Conventional MRI features, such as the T2-FLAIR mismatch sign and enhancement or necrotic patterns, show some association with IDH status [8]. However, these visually based approaches are experience-dependent, lack reproducible quantitative metrics, and demonstrate limited and unstable performance in predicting Ki-67 due to weak imaging–proliferation correlations [9–11]. Consequently, both clinical practice and research are shifting toward data-driven and quantitatively robust imaging analysis methods.

The emergence of deep learning, particularly convolutional neural networks (CNNs), has substantially advanced the ability to overcome the limitations of visually based imaging analysis. CNNs can automatically learn hierarchical and abstract feature representations from raw MRI data in an end-to-end manner, enabling the identification of complex imaging patterns that are difficult to capture through conventional approaches. In recent years, advanced CNN architectures such as ConvNeXt, which integrate design concepts from vision Transformers, have demonstrated excellent performance in medical image classification and segmentation owing to their strong representational capacity and computational efficiency [12]. Several studies have applied CNNs to multi-sequence MRI using early or late fusion strategies for IDH status prediction, highlighting the importance of multimodal information integration [13]. Other work has combined deep learning–based feature extraction with explainable artificial intelligence, such as SHapley additive explanations (SHAP), to accurately predict glioma pathological grade and Ki-67 proliferation level from T2-FLAIR images [14]. However, most existing studies treat IDH mutation and Ki-67 expression as independent prediction targets, despite their intrinsic biological association. Research employing multi-task learning frameworks to jointly model shared representations of IDH and Ki-67 remains limited, although such approaches are expected to improve model generalization and predictive performance by enabling knowledge sharing across tasks [15]. In addition, the lack of intuitive interpretability in many current models hampers clinician understanding and trust, posing a major barrier to clinical translation [16,17]. Therefore, developing a reliable, non-invasive approach for the simultaneous preoperative assessment of IDH and Ki-67 status remains of considerable clinical importance.

In this study, we developed and validated a ConvNeXt-Tiny–based multi-task deep learning model for non-invasive, simultaneous prediction of IDH mutation and Ki-67 status in gliomas using conventional multi-sequence MRI. The model was compared with single-task, radiomics, and clinical models, and its decision-making process was interpreted using SHAP.

## Materials and methods

### Inclusion and exclusion criteria

This retrospective study included adult patients with diffuse glioma treated at the First Hospital of Qinhuangdao (Center A, 01/01/2016–31/12/2025) and Baoding No.1 Central Hospital (Center B, 01/01/2018–31/12/2025). Inclusion criteria were: age ≥ 18 years, histopathologically confirmed diffuse glioma with WHO (2021) classification, known IDH1 mutation status and Ki-67 index¹⁸, first-time diagnosis with no prior tumor-related treatment, completion of high-quality preoperative multimodal MRI within 4 weeks before surgery (T2WI, T2-FLAIR, T1CE, 3D-ASL), and complete baseline clinical data (age, sex, preoperative KPS). Exclusion criteria included recurrent/secondary glioma, incomplete or poor-quality MRI, and prior intracranial surgery, radiotherapy, or conditions affecting brain MRI (e.g., extensive infarction or encephalitis). A total of 243 patients from Center A formed the training set, and 179 from Center B served as an independent validation set. The inclusion/exclusion workflow is shown in S1 Fig.

### Acquisition of clinical and pathological data

Clinical and pathological data were collected by two neurosurgeons following a standardized procedure. Clinical information, including age, sex, preoperative KPS score, and tumor location, was extracted from the electronic medical record system and cross-checked. Histological grades were recorded based on postoperative pathological reports and were strictly reviewed and reclassified according to the WHO Classification of Tumors of the Central Nervous System (2021) [18]. Key molecular markers, including IDH mutation status and the Ki-67 proliferation index, were obtained from standardized molecular pathology reports issued by the hospital pathology department. Ki-67 expression was categorized into low expression (≤10%) and high expression (>10%) groups using a threshold of 10%, based on established consensus in neuro-oncology and relevant evidence-based studies [19]. All data were independently entered and verified by two investigators, and any discrepancies were resolved by consultation with a third senior physician to reach consensus.

### Ethical approval and data access period

The authors accessed the data for research purposes from 07/01/2026–11/01/2026. Imaging data were obtained from the Picture Archiving and Communication System (PACS), and clinical data were accessed from the electronic medical record (EMR) systems of the First Hospital of Qinhuangdao and Baoding No.1 Central Hospital. All data were fully anonymized and de-identified prior to analysis to protect patient privacy. The study was approved by the Ethics Committee of the First Hospital of Qinhuangdao on 17/05/2023 (Approval No. 2023KZ065). The requirement for informed consent was waived due to the retrospective nature of the study.

### MR scanning

All multimodal magnetic resonance imaging data were acquired using standardized clinical scanning protocols. Preoperative examinations were performed on 3.0 T and 1.5 T magnetic resonance scanners, with specific model information provided in S1 Table. The acquired sequences included T2WI, T2-FLAIR, T1CE obtained after intravenous administration of a gadolinium-based contrast agent via the antecubital vein, diffusion-weighted imaging, and 3D-ASL. Scanning parameters followed the routine clinical imaging protocols of each participating center, with a uniform slice thickness of 5 mm and coverage of the entire brain. Detailed scanning parameters for each sequence are listed in S1 Table.

### Magnetic resonance image preprocessing workflow

**Fig 1:**
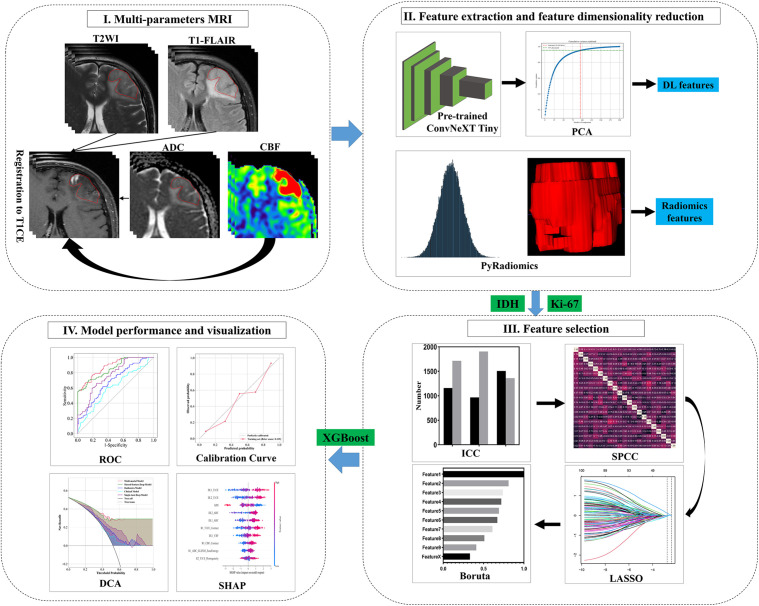
This flowchart presents a technical process for predicting glioma IDH mutations and Ki-67 proliferation index based on multi-parameter MRI. Step I. Multicontrast MRI preprocessing. The collected T2WI, T2-FLAIR, ADC, CBF and other multi-parameter MRI sequence images are registered (uniformly to T1CE spatial coordinates). **Step II.** Feature extraction and feature dimensionality reduction. Deep learning features are extracted through the pre-trained ConvNeXt Tiny model (after PCA dimension reduction) and radiomics features are extracted using PyRadiomics. **Step III.** Feature selection. Effective feature subsets are selected through ICC, SPCC, LASSO regression and Boruta algorithm for IDH and Ki-67 prediction. **Step IV.** A model is constructed using XGBoost, and its efficacy is verified through ROC curve, calibration curve and DCA, and the feature contribution degree is analyzed using SHAP plot.

The overall analysis workflow is summarized in Fig 1. All MRI data underwent a standardized preprocessing procedure to ensure comparability and accuracy for subsequent feature extraction and analysis. Preprocessing was performed in a Python (version 3.8) environment using NiBabel (3.2.1), SimpleITK (2.1.1), and ANTsPy (0.2.7). First, original DICOM images from each sequence, including T2WI, T2-FLAIR, T1CE, diffusion-weighted imaging, and ASL, were converted to NIfTI format. N4ITK bias field correction was then applied to each sequence to correct intensity inhomogeneity. All remaining sequences were rigidly registered to the T1CE images to achieve voxel-level spatial alignment. Each sequence was subsequently normalized using Z-score normalization to achieve zero mean and unit variance, thereby reducing inter-scanner variability. Finally, all images were resampled to a resolution of 1 × 1 × 5 mm³ and cropped to a fixed matrix size of 256 × 256 × Z, where Z represents the number of slices, to cover the entire brain.

Traditional radiomics features were extracted using the PyRadiomics 2.2.0 toolkit in Python. The extracted features included 8 first-order features, 18 two-dimensional shape features, 24 gray level co-occurrence matrix features, 16 gray level size zone matrix features, 16 gray level run length matrix features, 5 neighboring gray tone difference matrix features, and 14 gray level dependence matrix features. In total, 101 radiomics features were obtained for each imaging sequence.

### CNN network and training

This study employs the ConvNeXt-Tiny architecture to construct a dual-task deep learning model for simultaneous prediction of IDH mutation status and Ki-67 expression level in gliomas. The model inputs consist of axial T2WI, T2-FLAIR, T1CE, ADC, and CBF, together with the corresponding tumor region masks. To make full use of the limited sample size and improve model robustness, all axial two-dimensional slices containing tumor tissue from each patient were treated as independent samples during training and validation. The workflow was implemented as follows. First, the original NIfTI images were converted to PNG format and uniformly resampled to match the required network input size. During training, real-time data augmentation was applied, including random translation, rotation, scaling, shear transformation, Gaussian noise addition, and blurring, to enhance the generalization capability of the model. Transfer learning was adopted by initializing the network with ConvNeXt-Tiny weights pretrained on the ImageNet dataset. The original classification head was replaced with a customized dual-head output structure, in which each task head contained an independent linear classification layer that produced binary predictions for IDH status (mutant or wild type) and Ki-67 expression (low or high). The network architecture is illustrated in S2 Fig.

The model was fine-tuned end to end using the AdamW optimizer, with an initial learning rate of 1e-4, a weight decay of 0.05, and a batch size of 16. Validation loss was monitored during training with an early stopping strategy using a patience of 15 epochs, and the model with the lowest validation loss was retained. All experiments were conducted using the PyTorch framework on a workstation equipped with an NVIDIA RTX 3090 GPU. During the feature extraction stage, the dual-classification head was removed, and the preprocessed images were forwarded through the trained network. Deep features were extracted from the final global pooling layer, yielding a 768-dimensional feature vector for each image, as shown in Fig 1. These deep features were subsequently used for joint analysis and model interpretation.

To aggregate slice-level deep features into a unified patient-level representation, a feature averaging strategy was adopted. For each patient, the arithmetic mean was calculated for each feature dimension across the N 768-dimensional feature vectors extracted from all N tumor-containing two-dimensional slices. This procedure generated a single representative 768-dimensional patient-level feature vector, which was used for all subsequent joint analyses and interpretability assessments.

### Feature dimension reduction and selection

To reduce feature dimensionality and address multicollinearity, principal component analysis (PCA) was first applied to standardized deep learning features from the training set. Components with cumulative explained variance >95% or eigenvalues >1 were retained. The test set was projected into the same low-dimensional space using the PCA model fitted on the training set, ensuring preprocessing consistency and avoiding information leakage. A multi-stage feature selection strategy was then implemented to enhance robustness and interpretability. First, intraclass correlation coefficient (ICC, two-way random effects, absolute agreement) was used to assess repeatability, retaining only features with ICC > 0.80 based on independent delineations by two radiologists. Second, Spearman correlation screened out features not significantly correlated with either target, and features with absolute inter-feature correlation >0.90 were pruned, retaining those more strongly associated with the target. Third, two LASSO regression models were applied for IDH and Ki-67 prediction, with optimal λ determined by ten-fold cross-validation to retain features with non-zero coefficients. Finally, the Boruta algorithm, a random forest–based wrapper method, was used to confirm feature importance and stability over 1000 iterations, reducing spuriously correlated features and improving model generalization [20].

### Model establishment

This study constructed and systematically compared multiple predictive models for IDH mutation and Ki-67 expression. For each task, five models were evaluated: (1) a multimodal model integrating clinical, radiomics, and deep learning features; (2) a shared-feature deep model from the dual-task framework; (3) a radiomics-only model; (4) a clinical-only model including age, sex, tumor location, and KPS; and (5) a single-task deep model using task-specific network features. To interpret the shared-feature deep model, gradient-weighted class activation mapping (Grad-CAM) was applied to generate heat maps highlighting imaging regions critical for classification decisions. The net reclassification index (NRI) was used to quantify the incremental predictive performance of the multimodal model relative to each baseline model in the independent validation set. NRI significance was assessed using 95% confidence intervals derived from 1000 bootstrap resampling iterations.

### Statistical analysis

Statistical analyses were performed using MedCalc 20.0.14 (MedCalc Software BVBA, Ostend, Belgium) and R software version 3.6.3 (https://www.r-project.org). For continuous variables with a normal distribution, independent-sample t tests were used, whereas the Mann-Whitney U test was applied for non-normally distributed data. For categorical variables, chi-square tests or Fisher’s exact tests were used as appropriate. A two-sided P value less than 0.05 was considered statistically significant. The discriminatory performance of the models was evaluated using the area under the receiver operating characteristic curve (AUC). The goodness of fit of the nomogram model was assessed using calibration curves and the Hosmer-Lemeshow test. The clinical net benefit of the models was evaluated using decision curve analysis (DCA).

## Results

### Baseline feature description of training and test sets

Table 1 presents the clinical and demographic characteristics of patients in the training set (n = 243) and the test set (n = 179). No statistically significant differences were observed between the two groups with respect to age, gender ratio, tumor location distribution (*P* = 0.835), or KPS score. In addition, the proportions of IDH mutation status and Ki-67 expression levels did not differ significantly between the two groups. The distribution of WHO grades (II, III, and IV) was also comparable. Overall, the training and test sets showed good comparability in terms of major clinical characteristics, providing a stable and reliable foundation for subsequent model construction and validation.

**Table 1:** Clinical characteristics of patients in different sets.

Characteristic	Training set (n = 243)	Test set (n = 179)	*P*
Age (year, mean±SD)	53.5 ± 18.5	55.2 ± 17.3	0.342
Gender (%)			>0.999
Male	122 (50.2%)	89 (49.7%)	
Female	121 (49.8%)	90 (50.3%)	
Location (%)			0.835
Frontal lobe	90 (37.0%)	65 (36.3%)	
Non-frontal lobe	67 (27.6%)	54 (30.2%)	
Multilobar	86 (35.4%)	60 (33.5%)	
Grade (%)			
II	53 (21.8%)	30 (16.8%)	0.365
III	42 (17.3%)	37 (20.7%)	
IV	148 (60.9%)	112 (62.6%)	
KPS	75.4 ± 13.0	74.6 ± 14.7	0.557
IDH (%)			0.137
Mutant	121 (49.8%)	85 (47.5%)	
Wild-type	122 (50.2%)	94 (52.5%)	
Ki-67 (%)			
Low expression	103 (42.4%)	75 (41.9%)	>0.999
High expression	140 (57.6%)	104 (58.1%)	

Note: KPS: Karnofsky Performance Status; IDH: Isocitrate Dehydrogenase; Independent samples t-test was applied in continuous variables. Chi-Squared test or Fisher’s exact test was applied in categorical variables.

### Clinical model feature selection

In the training set (n = 243), univariate and multivariate logistic regression analyses were performed on clinical variables associated with IDH status and Ki-67 expression levels to identify features suitable for inclusion in subsequent modeling (Tables 2, 3). For IDH status, univariate analysis indicated that age, tumor location, and multi-lobar involvement were significantly associated with IDH mutation status. Multivariate analysis further demonstrated that non-frontal lobe location (adjusted OR = 1.943, 95% CI: 1.010–3.738, *P* = 0.047) and multi-lobar involvement (adjusted OR = 1.911, 95% CI: 1.038–3.519, *P* = 0.037) were independent risk factors for IDH wild-type status, while age showed a modest increasing risk trend (adjusted OR = 1.020, 95% CI: 1.006–1.035, *P* = 0.006).

**Table 2:** Univariate and Multivariate Logistic Regression Analysis for IDH in the Training Set (N = 243).

Variables	Univariate OR (95% CI)	*P Value*	Adjusted OR (95% CI)	*P Value*
Age (per year increase)	1.020 (1.006, 1.035)	0.005	1.020 (1.006, 1.035)	0.006
Gender (Male vs. Female)	1.280 (0.774, 2.119)	0.336		
Location				
Frontal lobe	Ref.		Ref.	
Non-frontal lobe	2.089 (1.098, 3.975)	0.024	1.943 (1.010, 3.738)	0.047
Multilobar	1.808 (0.994, 3.289)	0.052	1.911 (1.038, 3.519)	0.037
KPS (per 10-point increase)	1.161 (0.951, 1.411)	0.145		

Note: IDH = isocitrate dehydrogenase; OR=odds ratio

**Table 3:** Univariate and Multivariate Logistic Regression Analysis for Ki-67 in the Training Set (N = 243).

Variables	Univariate OR (95% CI)	*P Value*	Adjusted OR (95% CI)	*P Value*
Age (per year increase)	0.996 (0.983, 1.010)	0.603		
Gender (Male vs. Female)	0.755 (0.453, 1.260)	0.282		
Location				
Frontal lobe	Ref.			
Non-frontal lobe	0.750 (0.394, 1.428)	0.381		
Multilobar	0.838 (0.459, 1.530)	0.565		
KPS (per 10-point increase)	0.494 (0.386, 0.632)	<0.001	0.494 (0.386, 0.632)	<0.001

Note: IDH = isocitrate dehydrogenase; OR=odds ratio

For Ki-67 expression levels, age, gender, and tumor location were not statistically significant (all *P* > 0.05). In contrast, the KPS score showed a significant negative association with high Ki-67 expression. For every 10-point increase in KPS, the risk of high Ki-67 expression decreased significantly (adjusted OR = 0.494, 95% CI: 0.386–0.632, *P* < 0.001), confirming KPS as an independent protective factor.

In summary, IDH status was primarily influenced by tumor location and multi-lobar involvement, whereas Ki-67 expression levels were mainly associated with the patient’s KPS score. These clinical variables formed the basis for feature selection in the subsequent construction of clinical models and multimodal fusion models.

### Model training convergence

During training, the multi-task model demonstrated a stable convergence pattern. The accuracy and loss curves for both the training and validation sets indicated that the model gradually stabilized in predicting IDH mutation status and Ki-67 expression levels (see S3 Fig). These results suggest that the training process was reliable and showed no evident signs of overfitting.

### Feature dimension reduction and selection

To reduce dimensionality while preserving key information, PCA was applied to deep learning features from T2WI, T2-FLAIR, T1CE, ADC, and CBF, retaining components explaining 95% of variance (108, 95, 160, 142, and 139 features, respectively). These were combined with 101 radiomics features per sequence for IDH and Ki-67 prediction. Feature selection using ICC, SPCC, LASSO, and Boruta retained 16 features for IDH—mainly from T1CE (6), ADC (4), and CBF (5)—and 9 features for Ki-67—mainly from T1CE (4), ADC (3), and CBF (2); no effective features were retained from T2WI or T2-FLAIR (S2 and S3 Tables). Selected features showed weak or no intercorrelation (S4 Fig).

### Multi-modal model performance analysis

**Fig 2:**
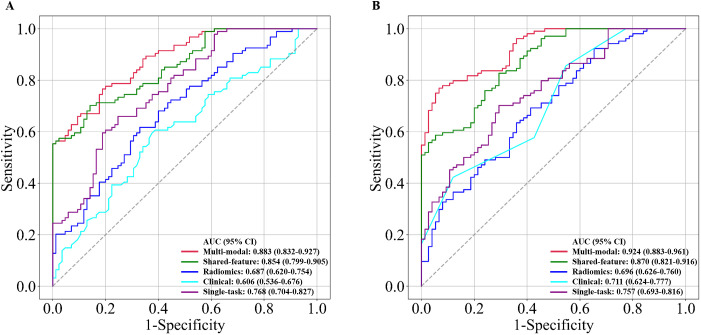
Receiver operating characteristic (ROC) curves of different models in the test set. **(A)** ROC for predicting IDH mutation status. **(B)** ROC for predicting high expression of Ki-67.

In the classification task of IDH mutation status, the multi-modal model demonstrated the best overall diagnostic performance (Fig 2, S4 Table). In the training set, the multi-modal model achieved an AUC of 0.901 (95% CI: 0.860 to 0.938) and maintained high accuracy (79.4%), sensitivity (78.7%), and specificity (80.2%). In the test set, the model continued to show stable performance with an AUC of 0.883 (95% CI: 0.832 to 0.927), outperforming the shared feature deep model (AUC = 0.854), the single-task deep model (AUC = 0.768), the radiomics model (AUC = 0.687), and the clinical model (AUC = 0.606). Further NRI analysis (S5 Table) demonstrated that the multi-modal model significantly improved the reclassification performance of cases in both the training and test sets. Compared with the radiomics model, the clinical model, and the single-task deep model, the multi-modal model achieved significantly positive NRI values in both datasets (training set NRI range: 0.975 to 1.296; test set NRI range: 0.985 to 1.248; all *P* < 0.01). When compared with the shared feature deep model, the multi-modal model also showed a significant reclassification advantage in the test set (NRI = 0.312, 95% CI: 0.033 to 0.597; *P* = 0.034). These results indicate that the multi-modal model has superior and more stable discriminative ability for IDH status prediction.

In the classification task of the Ki-67 proliferation index, the multimodal model also demonstrated the best diagnostic performance (Fig 2, S4 and S6 Tables). In the training set, the multimodal model achieved an AUC of 0.939 (95% CI: 0.909 to 0.967) and maintained the highest levels of accuracy (86.0%), sensitivity (85.7%), and specificity (86.4%). In the test set, the multimodal model continued to exhibit strong generalization performance with an AUC of 0.924 (95% CI: 0.883 to 0.961). Its specificity was markedly higher than that of the other models (93.3%), while it also maintained advantages in accuracy (83.8%) and F1 value (0.866). In comparison, the shared feature deep model, the single-task deep model, the radiomics model, and the clinical model showed notably lower AUC values, ranging from 0.757 to 0.870. Further NRI analysis (S7 Table) confirmed the significant advantage of the multimodal model in reclassification performance. In the training set, the multimodal model achieved significantly positive NRI values compared with the shared feature deep model, the radiomics model, the clinical model, and the single-task deep model (NRI range: 0.727–1.375, all *P* < 0.001). Consistent improvements were also observed in the test set (NRI range 0.882–1.196, all *P* < 0.01). These findings indicate that the multimodal model not only improved classification accuracy but also provided clear advantages in clinically relevant reclassification.

**Fig 3:**
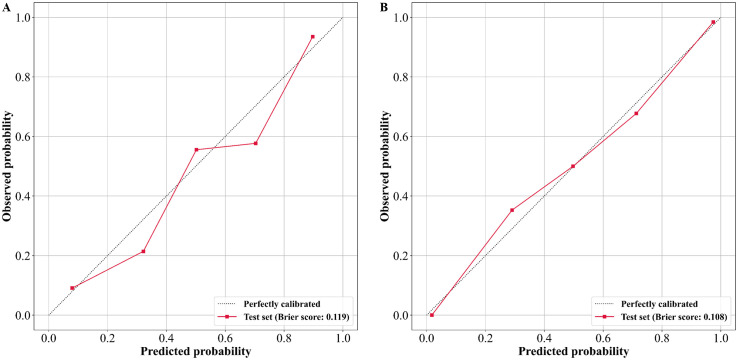
Calibration curves of different models in the test set. **(A)** Calibration curve for predicting IDH mutation status. **(B)** Calibration curve for predicting high expression of Ki-67. The dashed diagonal line represents perfect calibration, where predicted probabilities perfectly match observed outcomes. Closer proximity of a curve to this line indicates better model calibration.

**Fig 4:**
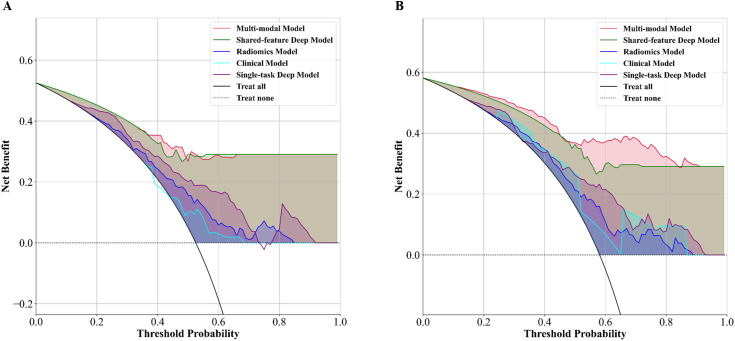
Decision curve analysis (DCA) evaluating the clinical utility of prediction models. **(A)** For IDH mutation prediction. **(B)** For high Ki-67 expression prediction.

Calibration curve analysis of the multi-modal model showed good agreement between predicted probabilities and observed outcomes. The Hosmer-Lemeshow test results were not statistically significant. For the IDH mutation status task, the P values for the training and test sets were 0.119 and 0.977, respectively, and for the Ki-67 proliferation index task, the corresponding P values were 0.152 and 0.455. These results indicate good model fit (Fig 3). Decision curve analysis demonstrated that the nomogram model was consistently positioned above the curves of the other models, indicating greater net clinical benefit for both prediction tasks (Fig 4).

### Model expalantion

**Fig 5:**
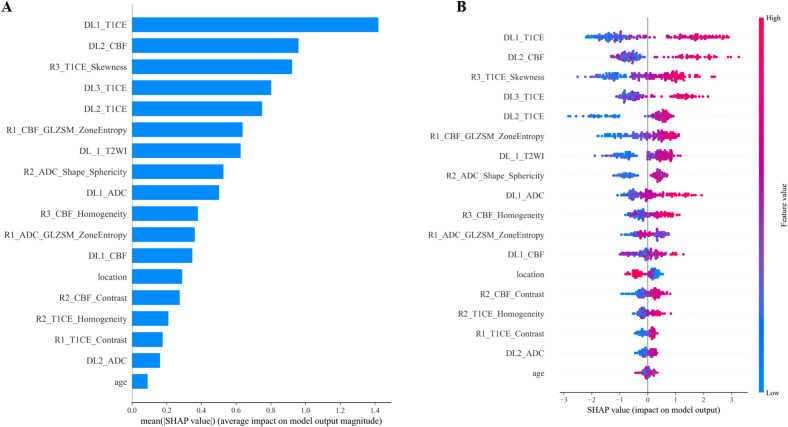
Feature importance and impact direction analysis of the multimodal model for predicting IDH mutation status. **(A)** Global feature importance ranking based on SHAP values. The bar length represents the mean absolute SHAP value for each feature, indicating its average magnitude of contribution to the model output. The plot integrates deep learning features (DL, derived from T1C, CBF, ADC, and T2WI sequences), radiomics features (R, derived from T1C, CBF, and ADC sequences), and clinical features (age, tumor location). **(B)** SHAP summary plot illustrating the direction and magnitude of feature effects. Each point represents an individual sample. The color of the point indicates the original feature value (red for high, blue for low), and its horizontal position (SHAP value) denotes the specific direction of that feature’s impact on the model prediction (positive SHAP values push the prediction toward IDH wild-type, while negative values push it toward IDH mutant).

For IDH prediction, the feature contribution ranking based on SHAP analysis showed that the multimodal model relied primarily on deep learning features (Fig 5). Among these, DL1_T1CE, DL3_T1CE, and DL2_T1CE from the T1CE sequence, as well as DL2_CBF from the CBF sequence, ranked highest and contributed most strongly to the prediction. Among radiomics features, R3_T1CE_Skewness from the T1CE sequence, R1_CBF_GLZSM_ZoneEntropy from the CBF sequence, and R2_ADC_Shape_Sphericity from the ADC sequence were included in the top 15 features. Among clinical variables, only Age and Location entered the top 15, with relatively smaller contributions. In terms of contribution direction, deep learning features such as DL1_T1CE and DL2_CBF showed positive effects on predicting IDH wild-type status by producing higher output values, whereas features such as R2_ADC_Shape_Sphericity showed negative effects.

**Fig 6:**
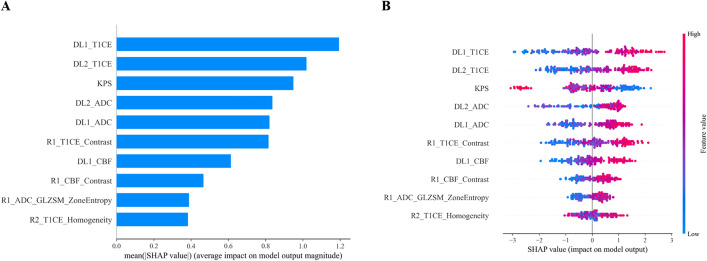
Feature importance and impact direction analysis of the multimodal model for predicting Ki-67 expression. **(A)** Global feature importance ranking based on SHAP values. The bar length represents the mean absolute SHAP value for each feature, indicating its average magnitude of contribution to the model output. The plot integrates deep learning features (DL, derived from T1C, CBF, ADC, and T2WI sequences), radiomics features (R, derived from T1C, CBF, and ADC sequences), and clinical features (KPS). **(B)** SHAP summary plot illustrating the direction and magnitude of feature effects. Each point represents an individual sample. The color of the point indicates the original feature value (red for high, blue for low), and its horizontal position (SHAP value) denotes the specific direction of that feature’s impact on the model prediction (positive SHAP values push the prediction toward Ki-67 high expression, while negative values push it toward Ki-67 low expression).

For Ki-67 prediction, the SHAP summary plot indicated that model predictions were mainly driven by deep learning features derived from multimodal MRI (Fig 6). Among these features, DL1_T1CE and DL2_T1CE had the highest absolute SHAP values and were the most influential contributors. These were followed by KPS, DL2_ADC, DL1_ADC, and R1_T1CE_Contrast. Deep learning features from perfusion imaging, particularly DL1_CBF, were also among the important contributors. Among radiomics features, R1_CBF_Contrast, R1_ADC_GLZSM_ZoneEntropy, and R2_T1CE_Homogeneity were included in the top 10, and their SHAP value distributions suggested stable contributions to the prediction task. From the perspective of feature influence direction, deep learning features from the T1CE sequence, including DL1_T1CE and DL2_T1CE, tended to show positive SHAP contributions at higher values, whereas deep learning features from the ADC sequence, such as DL1_ADC and DL2_ADC, were more likely to produce positive contributions at lower values. For radiomics features, higher ZoneEntropy values tended to increase the model output, while higher Homogeneity values generally showed negative contributions. In addition, the SHAP distribution of KPS was relatively concentrated, indicating that it mainly acted as a moderately positive contributing feature.

**Fig 7:**
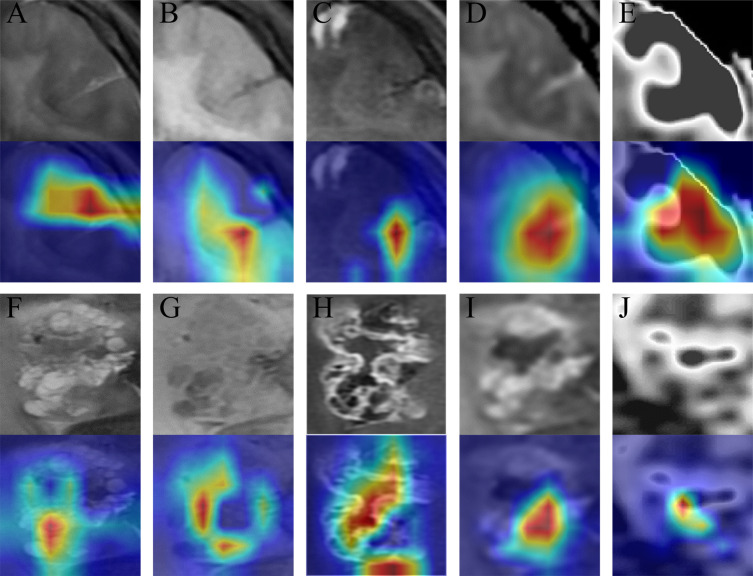
Grad-CAM visualization results for each sequence. This figure displays Grad-CAM visualization examples generated by the ConvNeXt-tiny model for individual images in the validation set. In the Grad-CAM heatmaps, warm-colored regions (red) indicate areas the model focuses on when making the corresponding classification. Panels A, B, C, D, and E show the original images and corresponding Grad-CAM activation maps for T2WI, T2-FLAIR, T1CE, ADC, and CBF sequences, respectively, from a patient with IDH-mutant and low Ki-67 expression. Panels F, G, H, I, and J show the original images and Grad-CAM activation maps for the same sequences from a patient with IDH-wildtype and high Ki-67 expression.

Based on the deep features from the last convolutional layer of the trained multi-task model, Grad-CAM visualization was performed on the test set. The results of superimposing the Grad-CAM heatmaps onto T2WI images are shown in Fig 7. The model assigned greater attention to the red regions and less attention to the blue regions. According to the Grad-CAM results, the multi-task model focused primarily on lesion tissue areas in a subset of test samples.

## Discussion

This study proposes a multi-task, multi-modal deep learning framework based on ConvNeXt-Tiny for simultaneous prediction of IDH status and Ki-67 levels in gliomas. By integrating five MRI sequences (T2WI, T2-FLAIR, T1CE, ADC, ASL-CBF) through a shared feature extraction module and combining imaging biomarkers with clinical features, the model jointly leverages structural, perfusion, and texture information across tasks. This shared feature mechanism enhances performance, outperforming radiomics, clinical, single-task, and non-multi-task deep learning models. SHAP-based analysis highlights the importance of shared features, particularly from T1CE, CBF, and ADC, supporting the biological plausibility of multimodal information for molecular and pathological prediction.

This study integrates radiomics features with CNN-based deep features and clinical indicators, achieving a balance between predictive performance and interpretability. While most existing studies prioritize deep learning features supplemented by radiomics, clinical variables often serve only as auxiliary factors. Prior work shows Swin Transformer-based models achieved AUCs of 0.868, multimodal fusion models 0.88–0.96, and T2-based deep learning 0.842 for IDH prediction [21,22]. Comparisons indicate high-level deep learning features outperform radiomics (AUC 0.915 vs. 0.830) [23], yet radiomics features remain valuable for interpretability, particularly in reflecting tumor shape and texture.

This study simultaneously predicted IDH status, reflecting molecular classification, and Ki-67 levels, representing tumor proliferative activity, within a single model, allowing a combined assessment of molecular subtype and malignancy. Earlier studies primarily focused on molecular markers such as IDH, 1p/19q, and TERT, with relatively few addressing Ki-67. Existing Ki-67 prediction models mostly relied on radiomics and traditional machine learning, achieving moderate performance (AUC 0.713–0.773) [24,25]. Some approaches used features from contrast-enhanced T1-weighted images with random forest classifiers (AUC 0.85, accuracy 80%) [26], while more recent models combined habitat subregions, deep learning, and clinical features, achieving high validation performance (AUC 0.950) but showing limited generalization in independent test sets [27]. Compared with these studies, our multimodal deep learning strategy integrates multiple MRI sequences and radiomics features, achieving strong predictive performance for both Ki-67 and IDH (validation AUC > 0.90 for Ki-67) while enhancing interpretability and generalization.

Recent studies have applied multi-task deep learning to jointly predict glioma molecular markers. For instance, a ViT-based TU-net model achieved high accuracy for IDH, MGMT, and Ki-67 but had limited sample size, lacked clinical features, and had low interpretability [28]. Another end-to-end multi-task network predicted IDH, 1p/19q, histological grade, and survival, outperforming single-task models for 1p/19q and prognosis but showing similar performance for IDH and grade [29]. These findings suggest that multi-task learning benefits some tasks through feature sharing but is not universally advantageous. In contrast, the present study focused on the biologically related dual-task combination of IDH and Ki-67 and demonstrated stable improvements in generalization and overall performance by integrating multimodal MRI and clinical features, highlighting that effectiveness depends on both task relevance and input information.

SHAP-based interpretability analysis identified the key deep learning and radiomics features driving the multimodal model and clarified the contributions of different MRI sequences to predicting IDH and Ki-67 status. For IDH prediction, deep features from T1CE sequences (e.g., DL1_T1CE, DL3_T1CE) and from the CBF sequence (DL2_CBF) contributed most to predicting wild-type status, suggesting that contrast enhancement patterns and tumor perfusion strongly influence IDH-related imaging phenotypes. This aligns with known characteristics of IDH wild-type tumors, which typically exhibit stronger enhancement, higher blood flow, and greater heterogeneity. Radiomics features, including T1CE_Contrast, ADC_Shape_Sphericity, and CBF_ZoneEntropy, also showed clear contribution directions, highlighting model sensitivity to tumor structural complexity, perfusion heterogeneity, and irregular morphology. For Ki-67 prediction, a similar pattern was observed: perfusion and contrast-enhanced sequences played major roles in characterizing tumor proliferative activity. These findings reinforce the biological plausibility of using multimodal MRI for molecular classification. ADC images, as noninvasive indicators, reflect cell density and biological behavior within the tumor microenvironment, serving as an important source for both IDH and Ki-67 prediction [30]. CBF-related features and ASL-derived perfusion images have been validated for distinguishing IDH and ATRX gene status [31–33], and correlate with Ki-67 expression and angiogenesis, as tumors with high proliferative activity often show increased blood flow [34]. Collectively, the SHAP analysis demonstrates that the multimodal model effectively integrates structural, perfusion, and texture information to capture biologically meaningful patterns underlying molecular and proliferative heterogeneity.

In this study, clinical characteristics such as age, tumor location, and KPS contributed to the multimodal model, but their SHAP values were substantially lower than those of deep learning and radiomics features, indicating a limited impact on overall predictive performance. Previous research has shown that patients with IDH wild-type gliomas are diagnosed at a higher median age (~59.2 years) compared to IDH-mutant patients (~39.0 years) [35], and frontal lobe tumors are more likely to harbor IDH mutations [36]. Choi et al. demonstrated that incorporating age and brain lobe location improved discrimination between IDH-mutant and wild-type patients [37], while Zhang et al. showed that combining imaging features with age and WHO grade enhanced the stability of IDH prediction [23]. For Ki-67, Liang et al. reported a significant association between expression levels and KPS (P = 0.017), suggesting a link between clinical functional status and tumor proliferative activity [38].

This study has several limitations. First, although multi-sequence MRI data were used, inconsistencies in acquisition parameters across some sequences may have influenced feature learning, and further improvement in multicenter standardization is needed. Second, a two-dimensional fusion strategy was adopted, which did not fully exploit three-dimensional volumetric information. Future studies may explore three-dimensional multimodal transformer architectures or diffusion-based models to enhance spatial representation. Third, although SHAP provides local explanations for feature importance, the specific biological significance of deep learning features requires further validation using pathological data. Finally, the sample size was relatively limited. Although the model showed stable performance in the test set, larger multicenter and prospective cohort studies are required to further confirm its clinical generalizability.

## Conclusion

In conclusion, this study developed a ConvNeXt-Tiny-based multi-task deep learning model for simultaneous preoperative prediction of IDH mutation and Ki-67 index in adult diffuse gliomas. The model showed robust performance, outperforming single-task deep learning, radiomics, and clinical models, demonstrating the value of multi-task architecture and multimodal data fusion for noninvasive molecular stratification.

## Supporting information

S1 Table Summary of Multicenter MRI Scanning Sequence Parameters. This table summarizes the MRI scanning parameters for each sequence used in this study.
(DOCX)

S2 Table Stepwise feature selection process for IDH mutation prediction. This table summarizes the number of candidate imaging features retained from each MRI modality at each feature selection step, including ICC assessment, Spearman correlation analysis, LASSO regression, and Boruta selection, for IDH mutation status prediction.
(DOCX)

S3 Table Stepwise feature selection process for Ki-67 expression prediction. This table summarizes the number of candidate imaging features retained from each MRI modality at each feature selection step, including ICC assessment, Spearman correlation analysis, LASSO regression, and Boruta selection, for Ki-67 expression prediction.
(DOCX)

S4 Table Diagnostic performance of different models for IDH mutation prediction. This table presents the diagnostic performance of the multimodal model, shared-feature deep model, radiomics model, clinical model, and single-task deep model for IDH mutation status prediction in the training and independent validation sets.
(DOCX)

S5 Table Net reclassification improvement analysis for IDH mutation prediction. This table compares the net reclassification improvement of the multimodal model relative to the shared-feature deep model, radiomics model, clinical model, and single-task deep model for IDH mutation status prediction across different datasets.
(DOCX)

S6 Table Diagnostic performance of different models for Ki-67 expression prediction. This table presents the diagnostic performance of the multimodal model, shared-feature deep model, radiomics model, clinical model, and single-task deep model for Ki-67 expression prediction in the training and independent validation sets.
(DOCX)

S7 Table Net reclassification improvement analysis for Ki-67 expression prediction. This table compares the net reclassification improvement of the multimodal model relative to the shared-feature deep model, radiomics model, clinical model, and single-task deep model for Ki-67 expression prediction across different datasets.
(DOCX)

S1 Fig Patient inclusion and exclusion workflow. (TIF)

S2 Fig Architecture of the ConvNeXt-Tiny multi-task learning model. Schematic diagram of a deep multi-task learning model architecture for the non-invasive prediction of glioma molecular markers. This figure visually illustrates the overall design workflow and core modules of the model. The left side shows the model input, featuring exemplary MRI images representing different molecular statuses (IDH-mutant/wild-type, Ki-67 low/high expression). The central part constitutes the model core, which is a deep convolutional neural network backbone. The hierarchical feature extraction process is indicated by the number of feature map channels (DMI = 96, 192, 384, 768), demonstrating the progressive abstraction from low-level to high-level semantic features. The right side displays the model output, showing the parallel prediction results for IDH status and Ki-67 expression level after processing by the task-specific fully-connected heads. This architecture achieves efficient co-prediction of multiple molecular markers by sharing low-level features while separating high-level decision-making.
(TIF)

S3 Fig Training and validation performance of the multi-task model for each MRI sequence. A–D: T2WI sequence, showing IDH prediction accuracy (A), IDH loss (B), Ki-67 prediction accuracy (C), and Ki-67 loss (D). E–H: T2-FLAIR sequence, showing IDH prediction accuracy (E), IDH loss (F), Ki-67 prediction accuracy (G), and Ki-67 loss (H). I–L: T1CE sequence, showing IDH prediction accuracy (I), IDH loss (J), Ki-67 prediction accuracy (K), and Ki-67 loss (L). M–P: ADC sequence, showing IDH prediction accuracy (M), IDH loss (N), Ki-67 prediction accuracy (O), and Ki-67 loss (P). Q–T: CBF sequence, showing IDH prediction accuracy (Q), IDH loss (R), Ki-67 prediction accuracy (S), and Ki-67 loss (T).
(TIF)

S4 Fig Heatmaps of features expressions in the (A) training set, and (B) external validation set, respectively. The bar represents Spearman correlation.
(TIF)

S1 File De-identified tabular data. This file contains the de-identified tabular data underlying the findings of this study, including cohort assignments, clinical variables, molecular labels, extracted radiomics features, deep learning features, model prediction signatures, and numerical data used to generate the figures and tables.
(ZIP)

## Abbreviations

AUC: Area under the curve
CNNs: Convolutional neural networks
DCA: Decision curve analysis
Grad-CAM: Gradient-weighted class activation mapping
IDH: Isocitrate dehydrogenase
LASSO: Least absolute shrinkage and selection operator
MRI: Magnetic resonance imaging
MTL: Multi-task learning
NRI: Net reclassification improvement
SHAP: Shapley additive explanations
T1WI: T1-weighted imaging
T1CE: Contrast-enhanced t1-weighted imaging
T2WI: T2-weighted imaging
T2-FLAIR: T2-weighted fluid-attenuated inversion recovery
XGBoost: Extreme gradient boosting
3D-ASL: Three-dimensional arterial spin labeling
