# Supplementary material for: A multimodal ConvNeXt-Tiny deep learning model for simultaneous prediction of IDH mutation and Ki-67 expression in gliomas
Source: PLoS One. 2026 Jun 26;21(6):e0351757. doi: 10.1371/journal.pone.0351757 (PMC13308780; doi:10.1371/journal.pone.0351757)
Supplement: S1 Table — This table summarizes the MRI scanning parameters for each sequence used in this study. (DOCX) [file pone.0351757.s001.docx]

**S1 Table. Summary of Multicenter MRI Scanning Sequence Parameters**

| Parameters | T2WI | T2-FLAIR | T1CE | DWI | ASL |
| --- | --- | --- | --- | --- | --- |
| MR scanner A（Center A, Siemens MAGNETOM Avanto 1.5T） | | | | |  |
| TR (ms) | 4400 | 8000 | 2300 | 3500 | 4861 |
| TE (ms) | 107 | 90 | 8 | 89 | 10.9 |
| Flip angle (°) | 120 | 150 | 120 | NA | NA |
| Slice thickness (mm) | 5 | 5 | 5 | 5 | 5 |
| Slice gap (mm) | 1.5 | 1.5 | 1.5 | 1.5 | 1.5 |
| Matrix | 256×256 | 256×256 | 320×320 | 192×192 | 384×384 |
| FOV (mm^2^) | 230×230 | 230×230 | 230×230 | 230×230 | 230×230 |
| b-value (s/mm^2^) | NA | NA | NA | 1000 | NA |
| MR scanner B（Center A, GE SIGNA Pioneer 3.0T） | | | | |  |
| TR (ms) | 4618 | 8000 | 3000 | 3739 | 4854 |
| TE (ms) | 120 | 90 | 24 | 80 | 10.7 |
| Flip angle (°) | 111 | 160 | 111 | NA | NA |
| Slice thickness (mm) | 5 | 5 | 5 | 5 | 5 |
| Slice gap (mm) | 1.5 | 1.5 | 1.5 | 1.5 | 1.5 |
| Matrix | 320×320 | 320×320 | 320×320 | 128×128 | 512×512 |
| FOV (mm^2^) | 240×240 | 240×240 | 240×240 | 240×240 | 240×240 |
| b-value (s/mm^2^) | NA | NA | NA | 1000 | NA |
| MR scanner C（Center B, Philips Achieva 1.5T） | | | | |  |
| TR (ms) | 4000 | 8000 | 2800 | 4000 | 4000 |
| TE (ms) | 120 | 100 | 20 | 80 | 12 |
| Flip angle (°) | 110 | 140 | 120 | NA | NA |
| Slice thickness (mm) | 5 | 5 | 5 | 5 | 5 |
| Slice gap (mm) | 1.5 | 1.5 | 1.5 | 1.5 | 1.5 |
| Matrix | 320×320 | 384×320 | 320×320 | 192×192 | 384×384 |
| FOV (mm^2^) | 240×240 | 240×240 | 240×240 | 240×240 | 240×240 |
| b-value | NA | NA | NA | 1000 | NA |

Note: T2WI: T2-weighted imaging; T2-FLAIR: T2-weighted fluid-attenuated inversion recovery; T1CE: T1-weighted imaging with contrast enhancement; DWI: diffusion-weighted imaging; ASL: arterial spin labeling; MR: magnetic resonance; TR: repetition time; TE: echo time; FOV: field of view
