# Supplementary material for: A multimodal ConvNeXt-Tiny deep learning model for simultaneous prediction of IDH mutation and Ki-67 expression in gliomas
Source: PLoS One. 2026 Jun 26;21(6):e0351757. doi: 10.1371/journal.pone.0351757 (PMC13308780; doi:10.1371/journal.pone.0351757)
Supplement: S2 Table — This table summarizes the number of candidate imaging features retained from each MRI modality at each feature selection step, including ICC assessment, Spearman correlation analysis, LASSO regression, and Boruta selection, for IDH mutation status prediction. (DOCX) [file pone.0351757.s002.docx]

**S2 Table. Stepwise feature selection process for IDH mutation prediction**

| Step | Method | T2WI | T2-FLAIR | T1CE | ADC | CBF | Total |
| --- | --- | --- | --- | --- | --- | --- | --- |
| 1 | Raw | 209 | 196 | 261 | 243 | 240 | 1149 |
| 2 | ICC | 165 | 158 | 203 | 192 | 180 | 898 |
| 3 | SPCC | 87 | 82 | 101 | 96 | 90 | 456 |
| 4 | LASSO | 8 | 6 | 10 | 6 | 7 | 37 |
| 5 | Boruta | 1 | 0 | 6 | 4 | 5 | 16 |

Note: IDH: isocitrate dehydrogenase; T2WI: T2-weighted imaging; T2-FLAIR: T2-weighted fluid attenuated inversion recovery; T1CE: T1-weighted contrast-enhanced; ADC: apparent diffusion coefficient; CBF: cerebral blood flow; ICC: intraclass correlation coefficient; SPCC: spearman rank correlation coefficient; LASSO: least absolute shrinkage and selection operator
