# Supplementary material for: A multimodal ConvNeXt-Tiny deep learning model for simultaneous prediction of IDH mutation and Ki-67 expression in gliomas
Source: PLoS One. 2026 Jun 26;21(6):e0351757. doi: 10.1371/journal.pone.0351757 (PMC13308780; doi:10.1371/journal.pone.0351757)
Supplement: S4 Table — This table presents the diagnostic performance of the multimodal model, shared-feature deep model, radiomics model, clinical model, and single-task deep model for IDH mutation status prediction in the training and independent validation sets. (DOCX) [file pone.0351757.s004.docx]

**S4 Table. Diagnostic performance of different models for IDH mutation prediction**

| Sets | Model | AUC (95% CI) | Acc (%) | Sen (%) | Spe (%) | Pre (%) | F1_max_ |
| --- | --- | --- | --- | --- | --- | --- | --- |
| Training | Multi-modal model | 0.901 (0.860-0.938) | 79.4 | 78.7 | 80.2 | 79.4 | 0.812 |
|  | Shared-feature Deep Model | 0.873 (0.831-0.914) | 79.0 | 71.3 | 86.8 | 84.5 | 0.782 |
|  | Radiomics Model | 0.668 (0.609-0.724) | 63.6 | 61.5 | 63.6 | 63.0 | 0.711 |
|  | Clinical Model | 0.631 (0.568-0.691) | 61.3 | 57.4 | 65.3 | 62.2 | 0.680 |
|  | Single-task Deep Model | 0.775 (0.720-0.827) | 77.5 | 64.8 | 81.0 | 77.5 | 0.733 |
| Test | Multi-modal model | 0.883 (0.832-0.927) | 78.8 | 76.6 | 81.2 | 81.8 | 0.808 |
|  | Shared-feature Deep Model | 0.854 (0.799-0.905) | 77.7 | 70.2 | 85.9 | 84.6 | 0.788 |
|  | Radiomics Model | 0.687 (0.620-0.754) | 64.2 | 61.7 | 67.1 | 67.4 | 0.718 |
|  | Clinical Model | 0.606 (0.536-0.676) | 60.9 | 59.6 | 62.4 | 63.6 | 0.704 |
|  | Single-task Deep Model | 0.768 (0.704-0.827) | 70.4 | 66.0 | 75.3 | 74.7 | 0.775 |

Note: AUC: area under the curve; CI: confidence interval; Acc: accuracy; Sen: sensitivity; Spe: specificity; Pre: precision
