# Supplementary material for: A multimodal ConvNeXt-Tiny deep learning model for simultaneous prediction of IDH mutation and Ki-67 expression in gliomas
Source: PLoS One. 2026 Jun 26;21(6):e0351757. doi: 10.1371/journal.pone.0351757 (PMC13308780; doi:10.1371/journal.pone.0351757)
Supplement: S5 Table — This table compares the net reclassification improvement of the multimodal model relative to the shared-feature deep model, radiomics model, clinical model, and single-task deep model for IDH mutation status prediction across different datasets. (DOCX) [file pone.0351757.s005.docx]

**S5 Table. Net reclassification improvement analysis for IDH mutation prediction**

| Comparison models | Data set | NRI (95% CI) | P |
| --- | --- | --- | --- |
| Multi-modal model vs. Shared-feature Deep Model | Training | 0.005 (-0.238-0.270) | 0.979 |
|  | Test | 0.312 (0.033-0.597) | 0.034 |
| Multi-modal model vs. Radiomics Model | Training | 0.975 (0.756-1.189) | <0.001 |
|  | Test | 1.007 (0.744-1.236) | <0.001 |
| Multi-modal model vs. Clinical Model | Training | 1.092 (0.905-1.291) | <0.001 |
|  | Test | 0.985 (0.785-1.189) | 0.005 |
| Multi-modal model vs. Single-task Deep Model | Training | 1.296 (1.097-1.468) | <0.001 |
|  | Test | 1.248 (1.015-1.470) | <0.001 |

Note: NRI: net reclassification index
