# Supplementary material for: A multimodal ConvNeXt-Tiny deep learning model for simultaneous prediction of IDH mutation and Ki-67 expression in gliomas
Source: PLoS One. 2026 Jun 26;21(6):e0351757. doi: 10.1371/journal.pone.0351757 (PMC13308780; doi:10.1371/journal.pone.0351757)
Supplement: S6 Table — This table presents the diagnostic performance of the multimodal model, shared-feature deep model, radiomics model, clinical model, and single-task deep model for Ki-67 expression prediction in the training and independent validation sets. (DOCX) [file pone.0351757.s006.docx]

**S6 Table. Diagnostic performance of different models for Ki-67 expression prediction**

| Sets | Model | AUC (95% CI) | Acc (%) | Sen (%) | Spe (%) | Pre (%) | F1_max_ |
| --- | --- | --- | --- | --- | --- | --- | --- |
| Training | Multi-modal model | 0.939 (0.909-0.967) | 86.0 | 85.7 | 86.4 | 89.6 | 0.876 |
|  | Shared-feature Deep Model | 0.869 (0.827-0.909) | 76.1 | 73.6 | 79.6 | 83.1 | 0.850 |
|  | Radiomics Model | 0.624 (0.564-0.683) | 60.9 | 60.7 | 61.2 | 68.0 | 0.743 |
|  | Clinical Model | 0.712 (0.654-0.770) | 65.4 | 62.1 | 69.9 | 73.7 | 0.755 |
|  | Single-task Deep Model | 0.783 (0.728-0.835) | 73.3 | 72.9 | 73.8 | 79.1 | 0.789 |
| Test | Multi-modal model | 0.924 (0.883-0.961) | 83.8 | 76.9 | 93.3 | 94.1 | 0.866 |
|  | Shared-feature Deep Model | 0.870 (0.821-0.916) | 77.7 | 82.7 | 70.7 | 79.6 | 0.842 |
|  | Radiomics Model | 0.696 (0.626-0.760) | 64.8 | 69.2 | 58.7 | 69.9 | 0.771 |
|  | Clinical Model | 0.711 (0.624-0.777) | 68.7 | 85.6 | 45.3 | 68.5 | 0.782 |
|  | Single-task Deep Model | 0.757 (0.693-0.816) | 70.4 | 70.2 | 70.7 | 76.8 | 0.797 |

Note: AUC: area under the curve; CI: confidence interval; Acc: accuracy; Sen: sensitivity; Spe: specificity; Pre: precision
