# Supplementary material for: A multimodal ConvNeXt-Tiny deep learning model for simultaneous prediction of IDH mutation and Ki-67 expression in gliomas
Source: PLoS One. 2026 Jun 26;21(6):e0351757. doi: 10.1371/journal.pone.0351757 (PMC13308780; doi:10.1371/journal.pone.0351757)
Supplement: S7 Table — This table compares the net reclassification improvement of the multimodal model relative to the shared-feature deep model, radiomics model, clinical model, and single-task deep model for Ki-67 expression prediction across different datasets. (DOCX) [file pone.0351757.s007.docx]

**S7 Table. Net reclassification improvement analysis for Ki-67 expression prediction**

| Comparison models | Data set | NRI (95% CI) | *P* |
| --- | --- | --- | --- |
| Multi-modal model vs. Shared-feature Deep Model | Training | 0.727 (0.504-0.952) | <0.001 |
|  | Test | 1.109 (0.862-1.353) | <0.001 |
| Multi-modal model vs. Radiomics Model | Training | 1.375 (1.178-1.568) | <0.001 |
|  | Test | 1.196 (0.958-1.440) | <0.001 |
| Multi-modal model vs. Clinical Model | Training | 1.204 (1.011-1.407) | <0.001 |
|  | Test | 1.091 (0.854-1.333) | 0.005 |
| Multi-modal model vs. Single-task Deep Model | Training | 0.919 (0.682-1.133) | <0.001 |
|  | Test | 0.882 (0.616-1.160) | <0.001 |

Note: NRI: net reclassification index
