# Supplementary figures and images for: A multimodal ConvNeXt-Tiny deep learning model for simultaneous prediction of IDH mutation and Ki-67 expression in gliomas
Source: PLoS One. 2026 Jun 26;21(6):e0351757. doi: 10.1371/journal.pone.0351757 (PMC13308780; doi:10.1371/journal.pone.0351757)

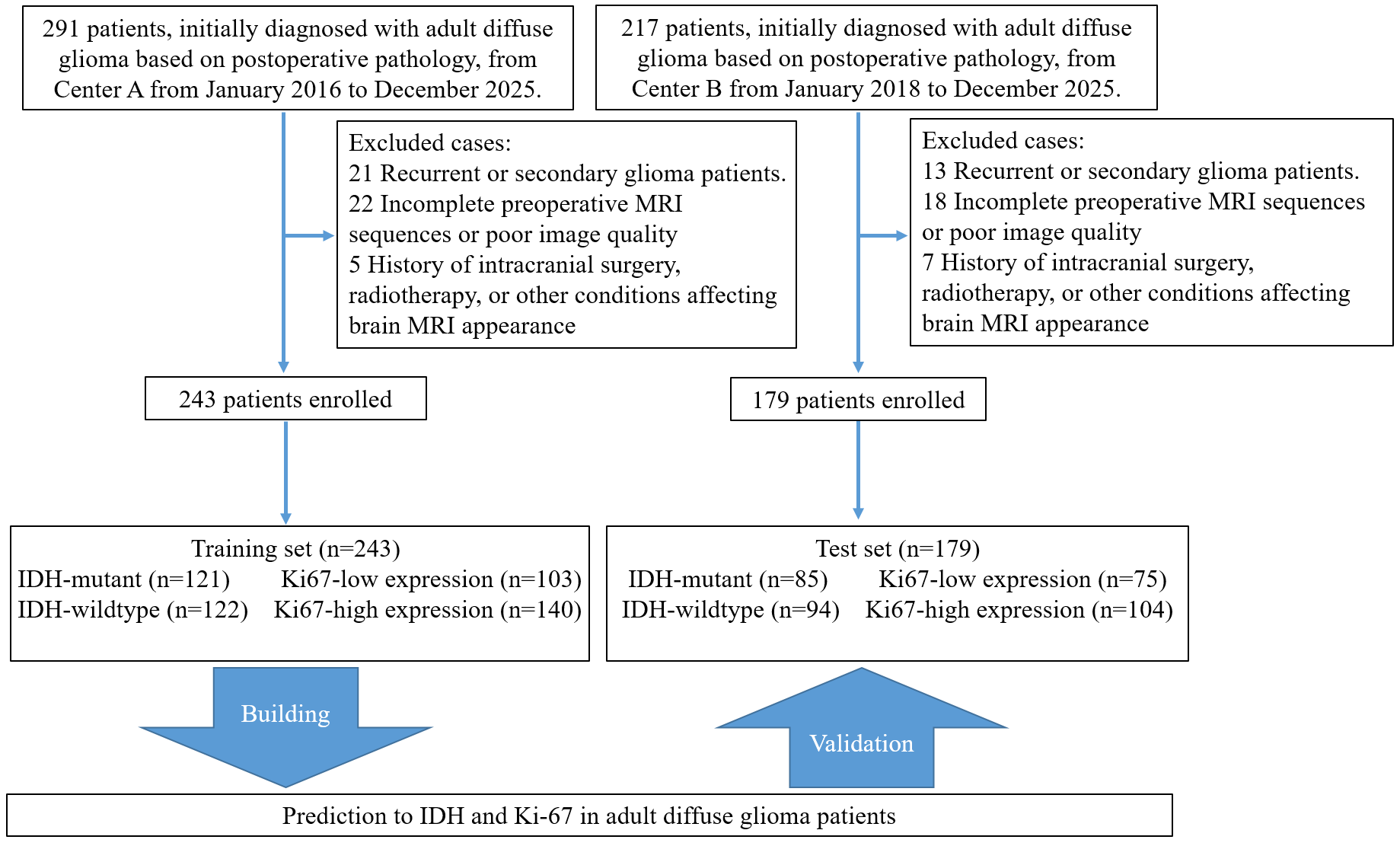

Supplement: S1 Fig — (TIF) [file pone.0351757.s008.tif]

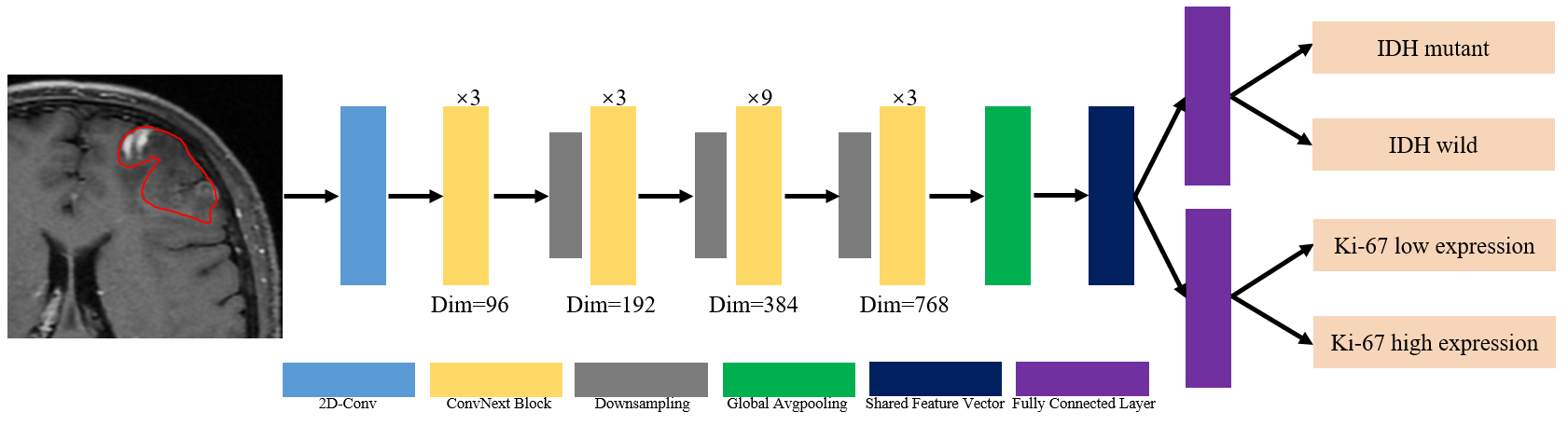

Supplement: S2 Fig — Schematic diagram of a deep multi-task learning model architecture for the non-invasive prediction of glioma molecular markers. This figure visually illustrates the overall design workflow and core modules of the model. The left side shows the model input, featuring exemplary MRI images representing different molecular statuses (IDH-mutant/wild-type, Ki-67 low/high expression). The central part constitutes the model core, which is a deep convolutional neural network backbone. The hierarchical feature extraction process is indicated by the number of feature map channels (DMI = 96, 192, 384, 768), demonstrating the progressive abstraction from low-level to high-level semantic features. The right side displays the model output, showing the parallel prediction results for IDH status and Ki-67 expression level after processing by the task-specific fully-connected heads. This architecture achieves efficient co-prediction of multiple molecular markers by sharing low-level features while separating high-level decision-making. (TIF) [file pone.0351757.s009.tif]

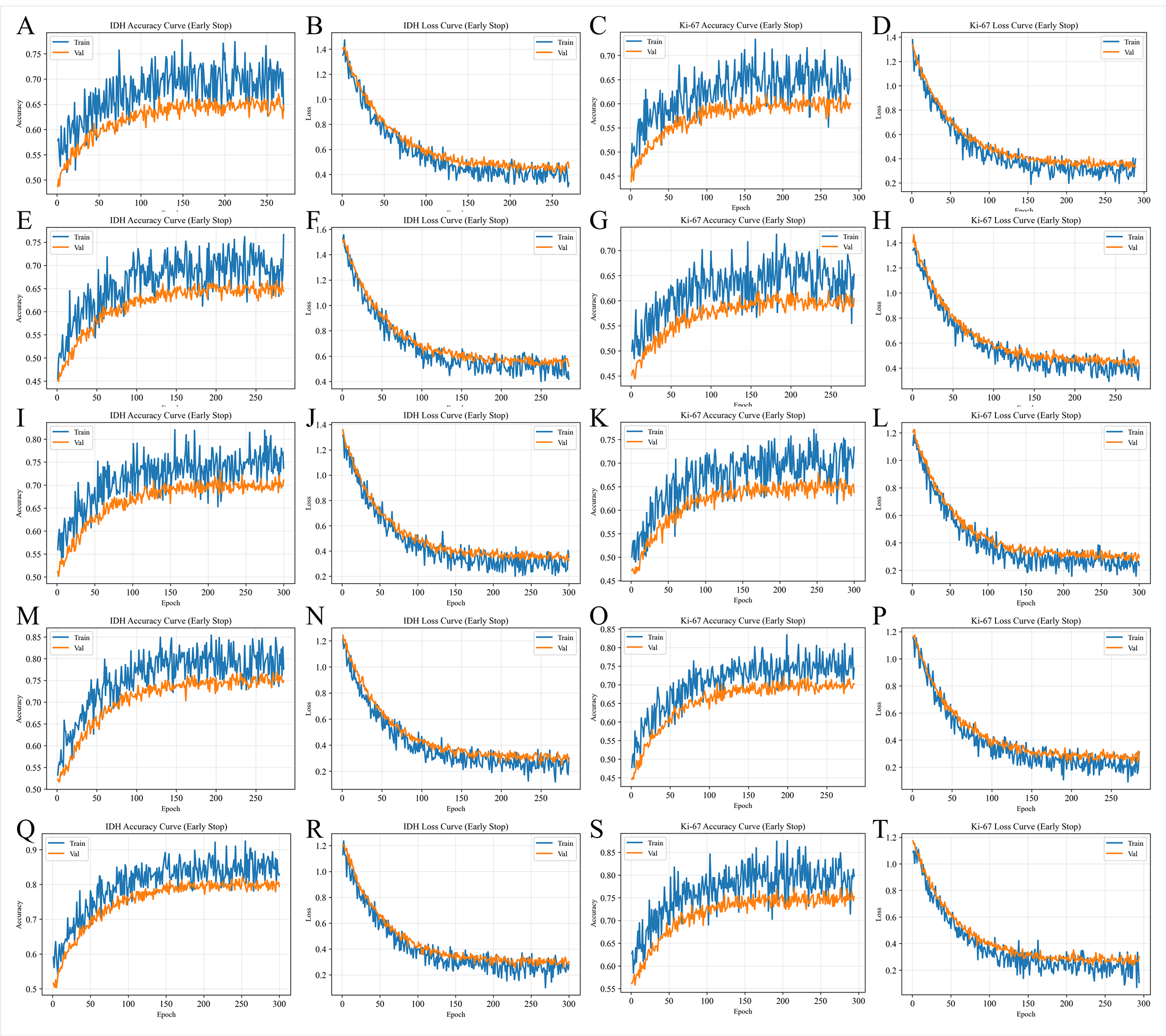

Supplement: S3 Fig — A–D: T2WI sequence, showing IDH prediction accuracy (A), IDH loss (B), Ki-67 prediction accuracy (C), and Ki-67 loss (D). E–H: T2-FLAIR sequence, showing IDH prediction accuracy (E), IDH loss (F), Ki-67 prediction accuracy (G), and Ki-67 loss (H). I–L: T1CE sequence, showing IDH prediction accuracy (I), IDH loss (J), Ki-67 prediction accuracy (K), and Ki-67 loss (L). M–P: ADC sequence, showing IDH prediction accuracy (M), IDH loss (N), Ki-67 prediction accuracy (O), and Ki-67 loss (P). Q–T: CBF sequence, showing IDH prediction accuracy (Q), IDH loss (R), Ki-67 prediction accuracy (S), and Ki-67 loss (T). (TIF) [file pone.0351757.s010.tif]

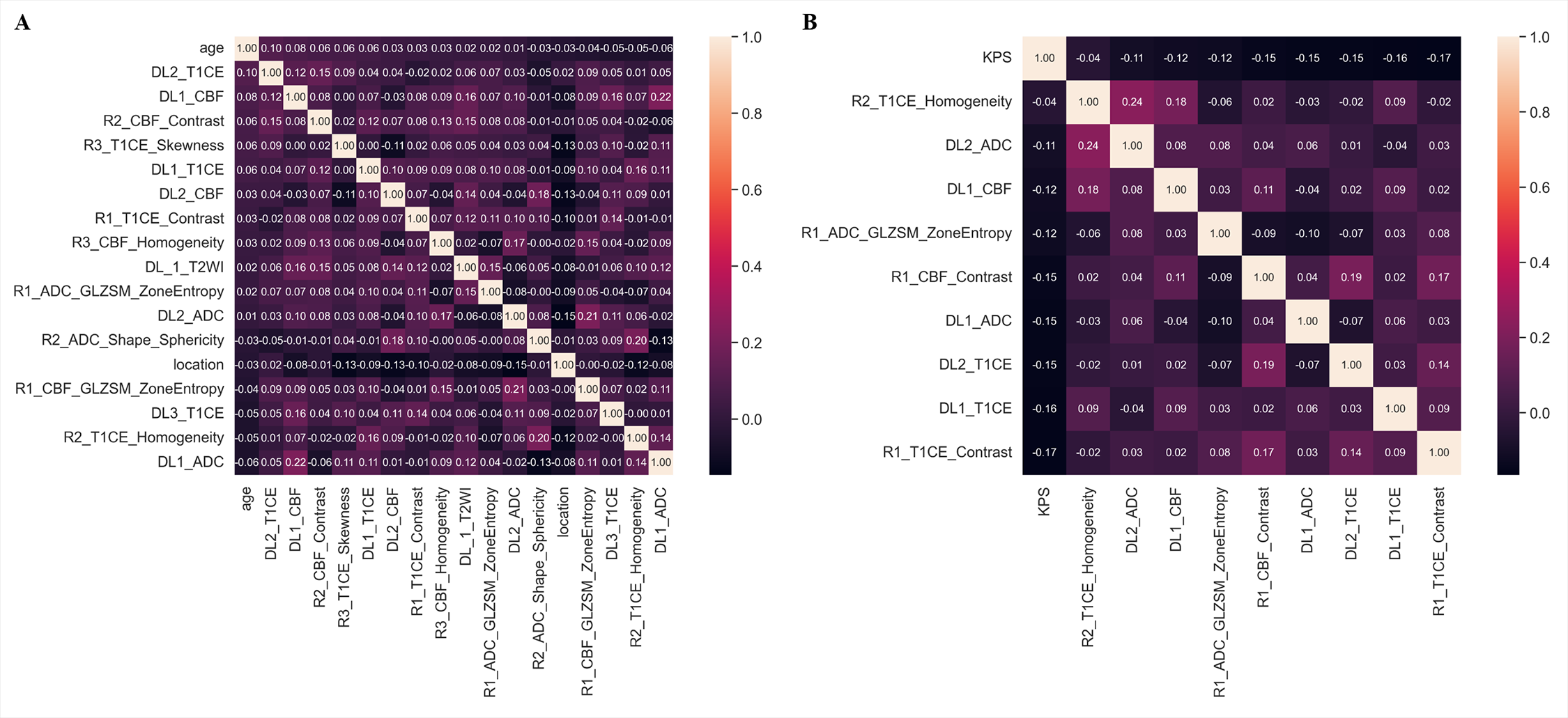

Supplement: S4 Fig — The bar represents Spearman correlation. (TIF) [file pone.0351757.s011.tif]
